# Emotion processing differences mediate the link between sex and autistic traits in young adulthood

**DOI:** 10.1002/jcv2.12096

**Published:** 2022-08-21

**Authors:** Lucy A. Livingston, Lucy H. Waldren, Esther Walton, Punit Shah

**Affiliations:** ^1^ Neuroscience and Mental Health Research Institute Cardiff University Cardiff UK; ^2^ Institute of Psychiatry, Psychology and Neuroscience King's College London London UK; ^3^ Department of Psychology University of Bath Bath UK

**Keywords:** alexithymia, autism spectrum disorder, emotion processing, mediation, sex differences

## Abstract

**Background:**

The male preponderance in autism diagnoses is widely reported, yet the psychological mechanisms (e.g., emotion processing) underlying this sex difference are poorly understood. Contributing to this gap in knowledge, most research has not been designed to investigate the intermediary (i.e., mediating) role of psychological processes in the relationship between sex and autism. Compounding this issue, concerns that autism measures are not reliably measuring the same constructs in males and females, and bias against females in clinical samples, make it difficult to investigate the psychological mechanisms underlying sex differences in autism.

**Methods:**

Over two cross‐sectional studies, 1656 young adults from the general population reported their sex (as assigned at birth) and completed questionnaires indexing their emotion processing differences, as well as a measure of autistic traits suggested to tap into the same psychometric construct in males and females.

**Results:**

Emotion processing differences mediated the relationship between sex and autistic traits, whereby being male was associated with more emotion processing differences, which were subsequently linked with greater levels of autistic traits. There remained a direct effect of sex on autistic traits after accounting for emotion processing differences.

**Conclusions:**

Emotion processing differences are a potential psychological mechanism underpinning higher prevalence of autism in males, which may serve a compensatory function in females; for example, females may seek out emotion‐inducing experiences to help compensate for social‐emotional difficulties. These findings inform our understanding of autism‐related sex differences and have potential implications for clinical practice, where the need for sex‐specific support and diagnostic processes is increasingly being recognised.

## Graphical abstract

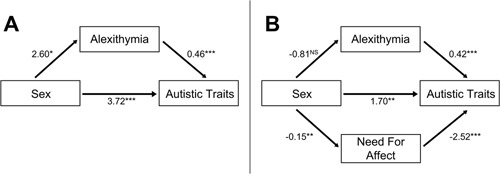

The male preponderance in autism diagnoses is widely reported, yet the psychological mechanisms underlying this sex difference is poorly understood. The present study investigated the mediating role of emotional processing differences in the relationship between sex (as assigned at birth) and autistic traits, in two large general population studies. Results showed that emotion processing differences mediated the relationship between sex and autistic traits, whereby being male was associated with more emotion processing differences, which were subsequently linked with greater levels of autistic traits.

**Key points**

- Autism is more commonly diagnosed in males (as assigned at birth) than females, but little is understood about the underlying psychological mechanisms.
- The present research investigated emotion processing differences as mediators in the relationship between sex at birth and autistic traits, in two large general population studies of young adults.
- Emotion processing differences partially mediated the relationship between sex and autistic traits, whereby being male was associated with lower need for affect, which was subsequently linked with greater levels of autistic traits. Alexithymia, however, did not robustly mediate the relationship between sex and autistic traits.
- The findings suggest that emotion processing differences may in part explain the male preponderance in autism diagnoses and highlight the possibility that need for affect may serve a compensatory function for females.

## INTRODUCTION

Autism Spectrum Disorder (hereafter autism) is a neurodevelopmental condition characterised by social communication differences and repetitive and restricted behaviours (American Psychiatric Association, 2013). One of the most well‐established features of autism is that males (as assigned at birth) are much more likely to receive a diagnosis than individuals born female (Lai et al., 2015). A recent meta‐analysis indicates a male:female ratio of around 3–4:1 in clinical diagnoses (Loomes et al., 2017) and males report more autistic traits than females in the general population (see Ruzich et al., 2015, for a systematic review). This evidence for a male preponderance in autism diagnoses remains robust, despite growing evidence to suggest under‐diagnosis of autism in females; for example, compared to males, females are diagnosed later (Begeer et al., 2013) and require more severe symptoms to be diagnosed (Dworzynski et al., 2012). However, even in studies that screen the population for autism—excluding studies that rely on existing clinical diagnoses, which can be biased against females—the ratio is still 3.25:1 (Loomes et al., 2017).

There is remarkably poor understanding of the mechanisms underlying the male preponderance in autism diagnoses. Most research has focused on biological explanations, which have received mixed empirical support; for example, the role of testosterone (Baron‐Cohen, 2002; but see Nadler et al., 2019) and sex‐specific genetic effects (Robinson et al., 2013; but see Bai et al., 2020). With this focus on biological correlates, possible psychological mechanisms, which can bridge links between complex biological constructs and behaviour (Frith, 2012), have been under‐investigated. Identifying intermediary psychological processes linking sex and autism also has potential to shed light on psychological sex differences, and as such, improve diagnostic precision for autism in males and females and inform sex‐specific clinical and educational support.

Several psychological mechanisms are atypical in autism, particularly with regards to social‐cognitive differences (Livingston & Happé, 2021). Emotion processing difficulties, for example, are consistently associated with autism and greater levels of autistic traits in the general population. This includes difficulties in recognising and discriminating basic and complex emotions (Black et al., 2017), understanding and experiencing others' emotional states (i.e., empathy; Shah et al., 2019), and identifying and labelling one's own emotions (known as alexithymia; Bird & Cook, 2013). Furthermore, studies have highlighted possible sex differences, whereby autistic males demonstrate more emotion processing difficulties than females, both on self‐report questionnaires and experimental tasks (see Harmsen, 2019). There are also longstanding reports of small‐to‐medium but highly significant sex differences in emotion processing in the general population (e.g., Greenberg et al., 2018; Lawrence et al., 2015; Saylik et al., 2018). Therefore, given the inter‐relationships between autism, sex, and emotion processing, a closer examination of emotion processing as a candidate psychological mechanism underpinning higher levels of diagnosed autism and autistic traits in males is needed.

One of the most widely documented emotion processing difficulties in autism is alexithymia, as observed in ∼50% of autistic people (compared to ∼5% of the general population; Kinnaird et al., 2019). Yet, little is known specifically about the role alexithymia plays in sex differences in autism and autistic traits, and there is conflicting evidence for sex differences in alexithymia in non‐clinical samples (e.g., Nam et al., 2020; Peng et al., 2019). Typically, studies have matched autistic and non‐autistic participants on age and sex (e.g., Milosavljevic et al., 2016) and sometimes alexithymia itself (e.g., Shah et al., 2016), to test whether alexithymia can help to explain mean differences between autistic and non‐autistic groups. To our knowledge, the relationship between alexithymia and autism‐related sex differences has not been directly investigated. Indeed, the latest meta‐analysis on alexithymia in autism was unable to draw any conclusions about the role of sex because of small and male‐skewed samples (typically <10 autistic females) in previous research (Kinnaird et al., 2019).

Despite major research interest in sex differences in emotion processing in autism, there are several outstanding issues to be addressed to investigate emotional processes in a manner that elucidates the association between sex and autism. First, the intermediary role of emotion processing in relation to greater levels of diagnosed autism and autistic traits in males than females is unclear. This is because research has focused on straightforwardly comparing groups of autistic males and females, rather than assessing the extent to which emotion processing mediates the relationship between sex and autism. Second, many autism measures, both diagnostic tools and trait questionnaires, are thought to be less sensitive to autism in females. As almost all measures were designed and validated with predominantly male samples, they may fail to measure autism as accurately in females as in males, thus making it difficult to study autism‐related sex differences (Lai & Szatmari, 2020). Third, compounding this problem, there is additional bias as to which types of females reach the clinic. For example, females without additional intellectual difficulty are less likely to be referred and subsequently diagnosed with autism (Carpenter et al., 2019), further undermining our confidence in previous sex‐based comparisons that have relied on clinically diagnosed samples. A general population trait‐based approach (see Happé & Frith, 2021), which overcomes clinical bias and non‐representative samples, and maximises statistical power, can be a powerful method for investigating sex differences in autism, especially when using trait measures of autism that are suggested to be less biased towards males (e.g., Grove et al., 2017). Additionally, that similar emotion processing differences have been found across clinical and general population samples (e.g., Kinnaird et al., 2019, but see Gregory & Plaisted‐Grant, 2016) also supports the use of a trait‐based approach to understand sex differences in autism, as in the current research.

### Current study

Addressing the challenges in previous research, we investigated emotion processing difficulties (in terms of alexithymia) as a psychological mediator in the relationship between sex (as assigned at birth) and autistic traits, in two large studies of young adults from the general population. Additionally, in Study 2, we utilised a well‐rounded, comprehensive approach to quantifying emotion processing differences, by measuring the motivation to engage in emotion‐inducing situations (need for affect), as well as difficulties in understanding one's own emotions (alexithymia). Importantly, across both studies, we used a measure of autistic traits that has been suggested to be equally sensitive to autistic traits across the sexes. Collectively, these studies represent the first investigation into the mediating role of emotional differences between sex and autistic traits in young adulthood.

## STUDY 1

### Methods and procedure

Six‐hundred and fifty‐six (515 females) young adults in the UK, aged 18–25 (*M*_age_ = 18.95, *SD*_age_ = 1.13), were recruited through local colleges and undergraduate university participant pools. Monte Carlo Power Analysis (using Schoemann et al., 2017) determined that we had 94% power to detect an indirect effect, given a small‐to‐medium standardised coefficient of 0.15 for each model path (*α* = 0.05, 5000 replications). After giving informed consent, participants completed three questionnaires in a randomised order, and reported their age and sex (as assigned at birth). Ethical clearance was granted from the local ethics committee.

Autistic traits were measured using the Autism‐Spectrum Quotient Short (AQ‐S; Hoekstra et al., 2011). Participants rate their (dis)agreement with 28 statements (e.g., “When I'm reading a story, I find it difficult to work out the characters' intentions”) using a 4‐point Likert scale from *Definitely Agree* to *Definitely Disagree*. Total possible scores range from 28 to 112, with greater scores indicating more autistic traits. The AQ‐S has demonstrated good internal consistency in general population samples (e.g., *α* = 0.82; Shah et al., 2019). Importantly, it has been shown to be invariant to sex in autistic individuals (i.e., it measures the same construct in autistic males [*n* = 265] and females [*n* = 285]; Grove et al., 2017), suggesting that it is equally sensitive to autistic traits in males and females in the wider population. In Study 1, the AQ‐S had good internal consistency (overall sample, *α* = 0.85; males, *α* = 0.81; females, *α* = 0.85).

Emotional difficulties—specifically, individual differences in alexithymia (i.e., difficulties identifying and describing one's own emotions)—were measured using the 20‐item Toronto Alexithymia Scale (TAS‐20; Bagby et al., 1994). Participants rate their (dis)agreement with 20 statements (e.g., “I am often confused about what emotion I am feeling”) on a 5‐point Likert scale from *Strongly Agree* to *Strongly Disagree.* Total possible scores range from 20 to 100, with greater scores indicating more alexithymia. The TAS‐20 has been widely used in general population samples, demonstrating excellent internal consistency (e.g., Shah et al., 2019, *α* = 0.90) and measurement invariance to sex (Peng et al., 2019). In Study 1, the TAS‐20 had good internal consistency (overall sample, *α* = 0.86; males, *α* = 0.83; females, *α* = 0.87).

#### Statistical analysis

Statistical analyses were conducted in JASP version 0.13.1 and *R* Studio version 4.1.1 (R Core Team, 2021). Mediation analyses were conducted using lavaan software (Rosseel, 2012). First, correlational analyses assessed the inter‐relationships between all variables. Second, a simple mediation analysis was conducted: Sex (1 = male, 0 = female) was entered as the independent variable, alexithymia as the mediator, and autistic traits as the dependent variable. Participant age was included as a covariate, given previous research on the interrelationships between age, sex, autism and alexithymia (Mattila et al., 2006; Tillman et al., 2018) and a difference in age between males and females in our sample, *t* (187.42) = −2.82, *p* = .005, *d* = 0.31 (males, *M* = 19.23, *SD* = 1.36; females, *M* = 18.88, *SD* = 1.05). Specifically, this model tested if alexithymia mediated the relationship between sex and autistic traits whilst accounting for age. Bias‐corrected percentile bootstrapping (10,000 re‐samples) estimated 95% confidence intervals (CIs) around total, direct, and indirect effects. All effects reported below are unstandardised.

### Results and discussion

There was a wide range of scores in autistic traits (30–96; *M* = 59.24, *SD* = 10.56) and alexithymia (23–82; *M* = 47.94, *SD* = 12.35), in line with previous research in non‐clinical samples (e.g., Shah et al., 2019). Autistic traits and alexithymia were positively correlated, *r*_*s*_ = 0.54, *p* < .001. Compared to females, males had significantly higher autistic traits, *r*_*s*_ = 0.20 *p* < .001 (males, *M* = 63.25, *SD* = 10.06; females, *M* = 58.14, *SD* = 10.43) and alexithymia, *r*_*s*_ = 0.09, *p* = .019 (males, *M* = 49.95, *SD* = 11.56; females, *M* = 47.39, *SD* = 12.51). Males were older, *r*_*s*_ = 0.10, *p* = .007, but age was not significantly correlated with autistic traits, *r*_*s*_ = 0.07, *p* = .09, or alexithymia, *r*_*s*_ = 0.01, *p* = .88.

**FIGURE 1:**
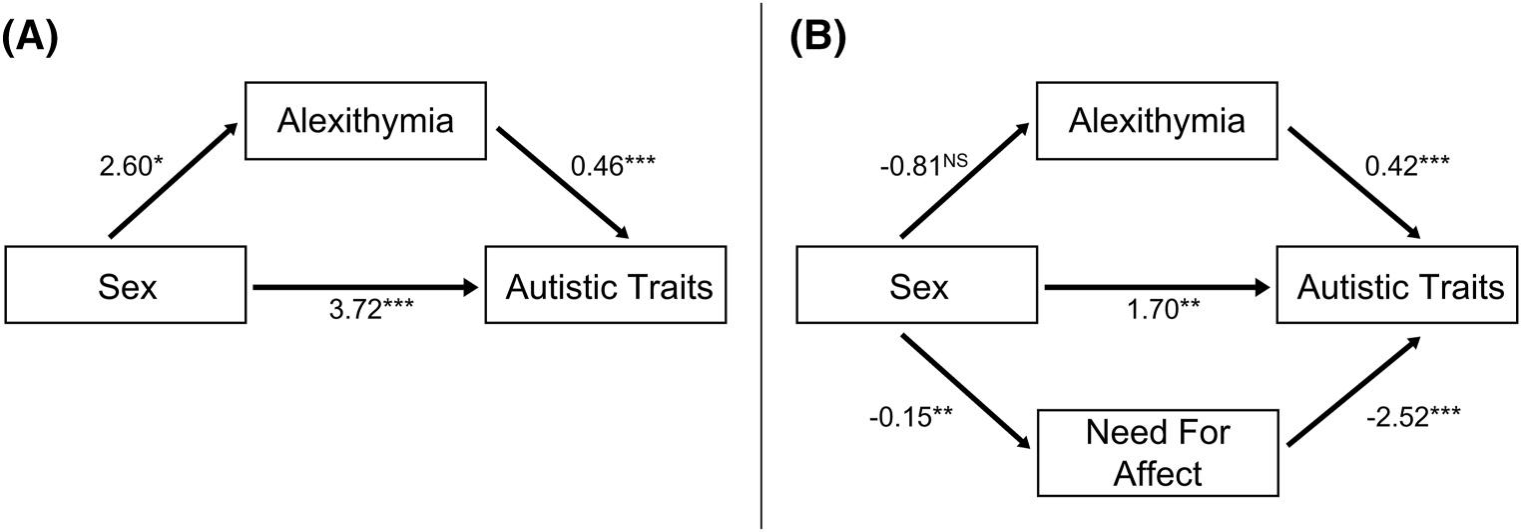
(A) The mediating role of alexithymia between sex and autistic traits, accounting for age and the direct path (Study 1). (B) The mediating roles of alexithymia and need for affect between sex and autistic traits, accounting for age, education level, and all other paths (Study 2). All coefficients are unstandardised. ***p < .001, **p < .01, *p < .05, NS, non‐significant

The mediation model is depicted in Figure 1A. Mediation analysis showed a significant overall effect of sex on autistic traits (total effect = 4.92, *SE* = 0.99, CIs [2.98–6.86], *p* < .001). When accounting for the indirect path via alexithymia, there remained a significant effect of sex on autistic traits (direct effect = 3.72, *SE* = 0.83, CIs [2.09–5.35], *p* < .001). Critically, when accounting for the direct path from sex to autistic traits, there was some evidence for mediation via alexithymia (indirect effect = 1.20, *SE* = 0.55, CIs [0.13–2.27], *p* = .029), whereby being male was associated with more alexithymia (*b* = 2.60, *SE* = 1.18, CIs [0.29–4.91], *p* = .027), which was subsequently associated with greater levels of autistic traits (*b* = 0.46, *SE* = 0.03, CIs [0.41–0.51], *p* < .001). *R* ^*2*^ indicated that the overall mediation model explained 33.1% of variance in autistic traits, *F*(3, 652) = 107.60, *p* < .001. This was a significant and substantial increase of 28.8% from a model only including sex and age as predictors of autistic traits, where 4.3% of variance was explained, *F* **Δ**(1, 652) = 281.00, *p* < .001. Overall, the results suggest partial mediation, whereby males showed greater levels of autistic traits, both directly and indirectly via alexithymia.

Study 1's findings suggest that alexithymia represents an autism‐related sex difference and thus, may be a possible psychological mechanism underpinning the male preponderance in autism diagnoses. That is to say, males may have more autistic traits partly due to increased difficulties in understanding their own emotions (see Cuve et al., 2022; Hobson et al., 2020 for further discussion). Study 1, however, had some limitations. First, it included a largely student and female sample, therefore our findings may not be generalisable to the wider young adult population. Second, the measure of alexithymia used in Study 1, the TAS‐20, has potential issues. It may measure psychological distress rather than alexithymia specifically (Preece et al., 2020) and its factorial validity has been questioned in both autistic and non‐autistic samples (e.g., Tuliao et al., 2020; Williams & Gotham, 2021).

Finally, and most fundamentally, Study 1 focused on alexithymia, that is, emotion processing difficulties, however there is a need for a more holistic understanding of emotion processing differences that could mediate autism‐related sex differences. Because most psychological autism research has focused on deficit‐based accounts, autism‐related differences have arguably been overlooked (Pellicano & den Houting, 2021; Taylor et al., 2022). Contrary to claims that autistic people are less attuned to their emotions, Smith (2009) proposes that some autistic people may experience emotions more strongly than non‐autistic people. For example, Shah et al. (2019) showed that autistic traits predicted greater levels of self‐reported emotional empathy (the ability to feel others' emotions) in a general population sample, after controlling for alexithymia and socio‐demographic variables. Additionally, an interaction between sex and alexithymia predicted emotional empathy, whereby the association between alexithymia and increased emotional empathy was significant in males but not females.

These findings raise the question as to whether certain aspects of emotion processing may be heightened in relation to autism, and subsequently play a role in autism‐related sex differences. One such emotion processing difference is ‘need for affect’; the tendency to approach and engage with emotion‐inducing situations and activities. This construct is widely studied in social psychology—for example, to understand individual differences (including sex differences) in social learning and behaviour (e.g., Appel et al., 2012; Bartsch et al., 2010; Haddock et al., 2008; Maio & Esses, 2001)—but has rarely been used in clinically‐relevant psychological science, especially compared to alexithymia. Whilst a sex difference in need for affect in the general population is widely reported, with males typically seeking fewer emotion‐inducing situations than females (e.g., Cramer et al., 2017; Leone & Presaghi, 2007; Maio & Esses, 2001), the construct has not been studied in relation to autism. Study 2, therefore, measured need for affect in addition to alexithymia, permitting a more comprehensive investigation into emotional processes mediating the link between sex and autistic traits.

## STUDY 2

Addressing the limitations of Study 1, we aimed to replicate and extend our findings in a large representative UK sample of 18‐25‐year‐olds. Specifically, we aimed to conduct a pre‐registered conceptual replication of Study 1, utilising a subset of 8 items from the TAS‐20 alexithymia measure that are purported to be equally, and potentially more, appropriate for studying autism‐related emotional difficulties (Williams & Gotham, 2021). Conceptual replications, where the same research question is addressed using a different methodology, are increasingly used in autism research to test the robustness of findings (e.g., Clutterbuck, Callan, et al., 2021; Taylor et al., 2021). Extending Study 1, to investigate the mediating role of emotion processing differences more comprehensively, we measured need for affect as well as alexithymia. Finally, we measured education level, which enabled us to assess the diversity of our sample, and subsequently account for this variable in analyses, given its previous link with emotion processing differences (e.g., Appel et al., 2012; Parker et al., 2005). In line with best practice, this study was pre‐registered prior to data collection (https://aspredicted.org/ZZP_63D).

### Methods and procedure

One thousand young adults, aged 18–25, were recruited via *Prolific* (487 females; *M*_age_ = 21.64, *SD*_age_ = 2.28). Participants were cross‐stratified by age and sex to reflect their representation in the UK population, according to Office for National Statistics 2020 data (e.g., 54 female 18‐year‐olds were recruited to match their 5.4% representation in the population). 12 additional participants were recruited but excluded for failing a basic attention check. Monte Carlo Power Analysis (Schoemann et al., 2017) determined that we had 99% power to detect indirect effects for two mediators, given a small‐to‐medium standardised coefficient of 0.15 for each path in the model (*α* = 0.05, 5000 replications).

Participants completed the same, reliable measure of autistic traits used in Study 1 (i.e., the AQ‐S; total sample, *α* = 0.80; males, *α* = 0.79; females, *α* = 0.81) and two measures of emotion processing differences, in a randomised order. The 8‐item General Alexithymia Factor Score (GAFS‐8; Williams & Gotham, 2021) contains 8 items from the TAS‐20 that most strongly reflect a “general alexithymia” factor and has demonstrated high reliability in autistic and general population samples (Williams & Gotham, 2021). Additionally, these 8 items require a lower reading level than the full TAS‐20 (Williams & Gotham, 2021), making the GAFS‐8 more suitable for representative general population samples, where education levels will vary. The GAFS‐8 5‐point Likert scale and total scoring is identical to the TAS‐20, therefore possible scores range from 8 to 40. In Study 2, the GAFS‐8 demonstrated good internal reliability (total sample, *α* = 0.86; males, *α* = 0.85; females, *α* = 0.87).

Need for affect (i.e., motivation to approach emotion‐inducing situations) was measured using the 10‐item Need for Affect Questionnaire Short (NAQ‐S; Appel et al., 2012). Participants respond using a 7‐point Likert scale (*Strongly Disagree* (−3) to *Strongly Agree* (3)), to 10 statements capturing either emotion approach (e.g., “I feel that I need to experience strong emotions regularly”) or avoidance (e.g., “I find strong emotions overwhelming and therefore try to avoid them”). Total scores are calculated as the mean across all 10 items, and range from −3 to +3, whereby positive scores reflect greater approach than avoidance (i.e., more need for affect). The NAQ‐S has demonstrated good internal consistency in general population samples (*α* = 0.82) and measurement invariance to sex (Appel et al., 2012). In Study 2, it showed good internal reliability (total sample, *α* = 0.79; males, *α* = 0.76; females, *α* = 0.82). Finally, participants provided information on their sex (as assigned at birth), age, and education level on a widely‐used 8‐point scale (0 = no formal qualifications, 7 = PhD; UNESCO Institute for Statistics, 2012).

#### Statistical analysis

A parallel mediation analysis was conducted, whereby two mediators (alexithymia and need for affect) were simultaneously modelled in the relationship between sex (1 = male, 0 = female) and autistic traits, with age and education level as covariates on all paths. Each mediation path controlled for the other in the model. Methods for bootstrapping were identical to Study 1 and effects are reported as unstandardised.

### Results and discussion

Wide‐ranging scores were found for autistic traits (*M* = 66.29, *SD* = 9.61), alexithymia (*M* = 23.45, *SD* = 7.14), and need for affect (*M* = 0.68, *SD* = 0.90). Additionally, the full range of education levels was represented (*M* = 3.27, *SD* = 1.69), demonstrating a more heterogenous sample compared to Study 1's largely student sample. Following a similar pattern as Study 1, autistic traits and alexithymia were positively correlated, *r*_*s*_ = 0.41, *p* < .001, and males reported greater levels of autistic traits, *r*_*s*_ = 0.10, *p* = .002 (males, *M* = 67.15, *SD* = 9.34; females, *M* = 65.39, *SD* = 9.81). However, there was no significant relationship between sex and alexithymia, *r*_*s*_ = −0.05, *p* = .10 (males, *M* = 23.07, *SD* = 6.87; females, *M* = 23.86, *SD* = 7.39). Need for affect was negatively correlated with autistic traits, *r*_*s*_ _*=*_ −0.38, *p* < .001, as well as sex, *r*_*s*_ = −0.09, *p* = .007, with males reporting a lower need for affect (males, *M* = 0.61, *SD* = 0.84; females, *M* = 0.76, *SD* = 0.95).

In terms of socio‐demographic variables, age was marginally associated with alexithymia, *r*_*s*_ = −0.06, *p* = .049, but not autistic traits, *r*_*s*_ = 0.01, *p* = .75, or need for affect, *r*_*s*_ = 0.03, *p* = .41. Education level was negatively correlated with alexithymia, *r*_*s*_ = −0.13, *p* < .001, and positively correlated with need for affect, *r*_*s*_ = 0.08, *p* = .007, but was unrelated to autistic traits, *r*_*s*_ = −0.04, *p* = .21, and sex, *r*_*s*_ = −0.02, *p* = .47. The correlations of age and education level with our independent, dependent, and mediator variables, underscore the importance of including these socio‐demographic variables as covariates.

The parallel mediation model is depicted in Figure 1B. In line with Study 1, a significant overall effect of sex on autistic traits was found (total effect = 1.74, *SE* = 0.60, CIs [0.56–2.92], *p* = .004). When accounting for the indirect paths via alexithymia and need for affect, there remained a significant effect of sex on autistic traits (direct effect = 1.70 *SE* = 0.54, CIs [0.64–2.75], *p* = .002). When accounting for the direct path from sex to autistic traits and the indirect path via need for affect, there was no significant mediation via alexithymia (indirect effect = −0.34, *SE* = 0.19, CIs [from −0.71 to 0.03], *p* = .08). This appeared to be driven by a lack of a sex difference in alexithymia (*b* = −0.81, *SE* = 0.45, CIs [from −1.68 to 0.07], *p* = .07), even though the path from alexithymia to autistic traits was significant (*b* = 0.42, *SE* = 0.04, CIs [0.33–0.50], *p* < .001).

There was significant mediation via need for affect, when controlling for all other paths in the model (indirect effect = 0.38, *SE* = 0.15, CIs [0.08–0.68], *p* = .012). Males had lower need for affect (*b* = −0.15, *SE* = 0.06, CIs [from −0.26 to −0.04], *p* = .008), which was subsequently associated with greater levels of autistic traits (*b* = −2.52, *SE* = 0.34, CIs [from −3.17 to −1.86], *p* < .001). *R* ^*2*^ indicated that the overall parallel mediation model explained 22.7% of variance in autistic traits, *F*(5, 994) = 58.39, *p* < .001, which represented a large 21.6% increase from a model with only sex, age, and education level as predictors of autistic traits, where 1.1% of variance was explained, *F* **Δ**(2, 994) = 138.82, *p* < .001. Overall, the findings indicate partial mediation, whereby being male (as assigned at birth) was associated with more autistic traits both directly and indirectly (via need for affect). This study provides the first evidence that need for affect may be a psychological mechanism underlying the male preponderance in autism diagnoses. Interestingly, this mediation was in a negative direction, that is, via reduced, rather than increased, need for affect.

The findings of Study 2 differed to those of Study 1; alexithymia did not significantly mediate the association between sex and autistic traits in Study 2 as in Study 1. When considering reasons for this difference, two methodological changes were identified. First, Study 2 included a larger, more diverse sample, with a nationally representative number of males and females. Second, there was a change in alexithymia measure from the TAS‐20 (Study 1) to the GAFS‐8 (Study 2). Although Study 2 was better powered and included an alexithymia measure designed for autism research, the discrepant results left it unclear whether alexithymia was genuinely a mediator of the link between sex and autistic traits.

To address this question, using the 8 items from the TAS‐20 that form the GAFS‐8, we (1) re‐conducted the mediation analysis of Study 1; and (2) pooled data across both studies to test alexithymia as a mediator, quantifying autistic traits (a) continuously, and (b) in terms of high versus low autistic traits using the AQ‐S clinical cut‐off. All analyses replicated the original findings of Study 2, converging to indicate that alexithymia is unlikely to mediate the association between sex and autistic traits (see ‘Additional Analyses’ in Supporting Information).

## GENERAL DISCUSSION

We investigated whether emotion processing differences mediated the relationship between sex and autistic traits in young adulthood. We found mixed evidence for the mediating role of alexithymia, whereby being male (as assigned at birth) was linked with greater levels of autistic traits via increased alexithymia in Study 1, but not Study 2. Furthermore, in Study 2, we found significant mediation via need for affect, whereby males reported lower need for affect, which was subsequently associated with greater levels of autistic traits. Overall, therefore, we report evidence that emotion processing differences, and most pertinently, lower need for affect, underpin sex differences in autistic traits, thus highlighting that these psychological processes may partly explain the male preponderance in autism diagnoses. Nevertheless, that a direct effect of sex on autistic traits remained in both studies, suggests sex differences in autism are not fully explained by emotion processing differences.

The present research was the first to investigate need for affect as a possible mechanism underlying autism‐related sex differences (Study 2). The established relationship was, however, in the direction of males having lower need for affect, leading to more autistic traits. This suggests that this specific emotion processing difference does not account for the male preponderance in autism diagnoses, and in fact, that females may typically report fewer autistic traits in part due to higher need for affect. Although tempting to reframe this finding as another autism‐related emotional difficulty in males, high need for affect may not always be advantageous. For example, individuals high in need for affect tend to be more influenced by emotions during information processing, which can detract from more cognitive‐based decision making (Haddock & Maio, 2019). Further, in individuals with high autistic traits, reduced motivation to engage in emotions in the face of social‐emotional challenges may be adaptive. Further research is needed to establish in which contexts need for affect might be a strength or difficulty relevant to sex differences in autism.

Alexithymia was not found to be a robust mediator of the relationship between sex and autistic traits. However, the present study highlights some interesting issues surrounding this psychological construct, which is increasingly used in autism research (e.g., Cook et al., 2013; Shah et al., 2016). It was previously unclear whether sex differences in alexithymia in relation to autism are robust given that most studies have included small numbers of females (Kinnaird et al., 2019). The current research, including over 1000 female participants, represents one of the largest alexithymia‐related studies on autism to date. It suggests that sex differences in alexithymia, at least in relation to autistic traits, may in fact be minimal and smaller than previously assumed. However, given the limited research into sex differences in alexithymia in the context of autism, further research is required. For example, using the GAFS‐8 measure alongside alternative measures of alexithymia that are completely independent from the TAS‐20 (e.g., the Bermond‐Vorst Alexithymia Questionnaire; Vorst & Bermond, 2001), as well as interview‐based alexithymia scales (Bagby et al., 2006; Sekely et al., 2018), may be useful to shed further light on autism‐related sex differences in alexithymia.

Our findings have potential implications for research and clinical practice. First, they build on previous research showing that emotion processing differences are highly relevant to understanding autism and autistic traits, and specifically, presentation amongst males. Emotion processing can be readily measured through brief self‐ or parent‐report questionnaires, which may benefit clinicians diagnosing young autistic people. It is nonetheless important for clinicians to consider that emotion processing is multi‐faceted and that not all facets will be universally implicated. For example, the present study highlights the role of need for affect, but not necessarily alexithymia, for understanding autism‐related sex differences. Measuring emotion processing using questionnaire‐based scales is also limited; people may lack insight into their own emotion processing skills (see Huggins et al., 2020; Marchesi et al., 2014; Petrides & Furnham, 2000). In addition to being a limitation of our study, this also represents a significant challenge for accurately measuring emotion processing in time‐restricted clinical settings.

Second, the findings suggest that autism‐related emotion processing differences are less pronounced in females compared to males. This is in line with broader research suggesting sex‐specific psychological profiles in autism (e.g., across social cognition, executive function, and memory; see Hull et al., 2017) and calls for greater appreciation of the impact of sex on the development, presentation, and diagnosis of autism (Lai & Szatmari, 2020). The findings also suggest the possibility that need for affect may serve a compensatory function for females. For example, females with a higher propensity for autism may seek out emotion‐inducing experiences to help compensate for  difficulties with social‐emotional processing. This could be explored further using multi‐level data (e.g., cognitive, behavioural, genetic), as required for the study of compensation (Livingston et al., 2021; Livingston & Happé, 2017).

Third, the findings highlight the importance of psychological, alongside more biological, mechanisms to explain the male preponderance in autism diagnoses. Across mediation models, a sizeable amount of variance in the relationship between sex and autistic traits (up to ∼30%) was explained by emotion processing. There are many other relevant psychological processes to explore, given their link with sex and autism, including other aspects of social cognition, such as theory of mind (Clutterbuck, Shah, et al., 2021), executive function (Demetriou et al., 2021), and social motivation (Sedgewick et al., 2016). Indeed, all our models showed a direct effect of sex on autistic traits, when controlling for emotion processing differences and sociodemographic factors, suggesting that many other psychological processes are likely contributing to the impact of sex on autistic traits. Moving forward, biological (e.g., genetics and epigenetics) and psychological (e.g., emotion processing, other facets of social and non‐social cognition) processes should be studied together to inform a more holistic understanding of sex differences in autism.

Our findings should be considered in light of some limitations. First, whilst we used a measure that has been suggested to be equally sensitive to autistic traits in males and females, arguably this measure captures a form of autism more likely to be observed in males. For example, it has been proposed that a ‘female autism phenotype’ exists, which shares core characteristics with the traditional conceptualisation of autism, but has some qualitative distinctions (e.g., increased social motivation, female‐specific special interests, and a greater tendency to use compensatory/camouflaging strategies; Hull et al., 2020; Livingston & Happé, 2017). Therefore, our findings may be less relevant to a possible female autism phenotype, although empirical evidence for its existence is currently lacking. Second, although a trait approach enabled well‐powered analyses and overcame issues with sex differences induced by clinical bias, results from autism trait research do not always translate into clinical observations (e.g., Fusar‐Poli et al., 2020; Keefer, 2015; Lumley et al., 2005). This is in part due to cognitive differences between individuals with autism and the general population samples with which these measures were developed (e.g., metacognition and theory of mind; Grainger et al., 2014; Huggins, 2020; Zalla et al., 2015). There is also ongoing debate about the extent to which autism can be conceptualized as quantitative traits versus a categorical, clinical approach to classification (Peralta & Cuestra, 2007; Volkmar & McPartland, 2016). As such, the findings now require replication in individuals with diagnosed autism. Second, future studies might benefit from collecting additional participant information (e.g., ethnicity) to ensure studies are representative of both autistic and general populations, as well as investigating if similar results are evident when measuring gender identity instead of sex. Finally, emotion processing differences are found in numerous other psychiatric conditions (e.g., anxiety, mood disorders, schizophrenia; see Kret & Ploeger, 2015 for a review), which often co‐occur with autism, but were not accounted for in our analyses. Further research should assess the specific intermediary role of emotion processing in the relationship between sex and autism, over and above additional mental health problems.

Overall, across two large general population studies of young adults, we found evidence that emotion processing differences—specifically, need for affect—mediated the link between sex and autistic traits, thus implicating them as possible mechanisms underlying male preponderance in autism diagnoses. Evidence for the role of alexithymia was mixed and requires further investigation. Future research should now investigate a range of intermediatory psychological and biological processes together, towards further elucidating the mechanisms underlying important sex differences in autism.

## AUTHOR CONTRIBUTIONS

Lucy Livingston: Conceptualization, Formal analysis, Supervision, Writing—original draft; Lucy Waldren: Data curation, Formal analysis, Writing—original draft; Esther Walton: Conceptualization, Supervision, Writing—review & editing; Punit Shah: Conceptualization, Data curation, Formal analysis, Funding acquisition, Supervision, Writing—review & editing.

## CONFLICT OF INTEREST

The authors have declared that they have no competing or potential conflicts of interest.

### OPEN RESEARCH BADGES

This article has been awarded Open Materials, Open Data, Preregistered Research Designs badges. Study 2 analyses were pre‐registered at https://aspredicted.org/ZZP_63D and materials, data and *R* code are publicly available in the Supporting Information of this article. Learn more about the Open Practices badges from the Center for Open Science: https://osf.io/tvyxz/wiki.

## ETHICAL CONSIDERATIONS

The study received full ethical approval from the local ethics committee.

## Supporting information

Supplementary Material

Supplementary Material

## Data Availability

The data and *R* code for analyses are available in the Supporting Information of this article.
