# Supplementary material for: Emotion processing differences mediate the link between sex and autistic traits in young adulthood
Source: JCPP Adv. 2022 Aug 21;2(3):e12096. doi: 10.1002/jcv2.12096 (PMC10242897; doi:10.1002/jcv2.12096)
Supplement: Supplementary file 1 — Supplementary Material [file JCV2-2-e12096-s001.docx]

**Supporting Information**

Livingston, L.A., Waldren, L.H., Walton, E., & Shah, P. (2022). Emotion processing differences mediate the link between sex and autistic traits in young adulthood. *JCPP Advances.*

**Additional Analyses**

We conducted additional analyses to assess the robustness of our findings pertaining to alexithymia as a mediator between sex and autistic traits. First, we re-conducted the mediation analysis of Study 1, quantifying alexithymia using the 8 items from the TAS-20 that form the GAFS-8 (as in Study 2). This finding supported Study 2; alexithymia did not mediate the association between sex and autistic traits (indirect effect = 0.81, *SE* = 0.50, CIs[-0.17–1.78], *p* = .10; Figure S1-A).

Second, to maximise statistical power, we pooled data across Studies 1 and 2 (*N* = 1656), again finding that alexithymia (i.e., in terms of GAFS-8) was not a significant mediator in the model (indirect effect = 0.29, *SE* = 0.25, CIs[-0.20–0.78], *p* = .24; Figure S1-B). In a final analysis of the pooled data, to further assess the robustness of our results, we tested whether alexithymia mediated the link between sex and group membership to high (*n* = 471) and low (*n* = 1185) autistic trait groups, based on the AQ-S clinical cut-off (≥ 70; Hoekstra et al., 2011). This analysis revealed further evidence that alexithymia was not a significant mediator (indirect effect = 0.01, *SE* = 0.01, CIs[-0.01–0.03], *p* = .25; Figure S1-C). Conceptually replicating Study 2, all additional analyses converged to indicate that alexithymia is unlikely to mediate the association between sex and autistic traits.

*
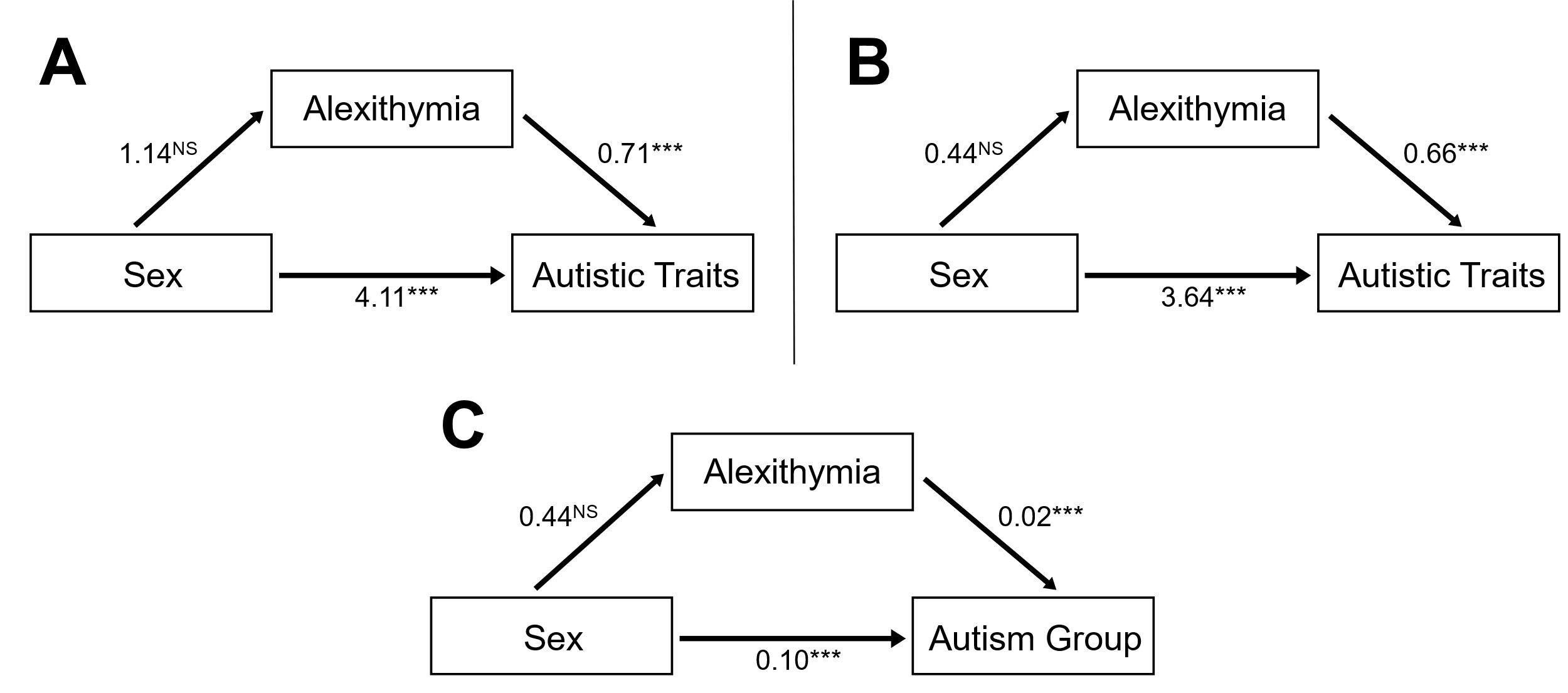
*

*Figure S1.* A: Additional analyses of Study 1 data using the GAFS-8 instead of the TAS-20, accounting for age and the direct path. B: Analysis of data pooled across Study 1 and 2 with a continuous measure of autistic traits. C: Analysis of data pooled across Study 1 and 2 with autism quantified as participants who met (high autistic trait group = 1) and did not meet (low autistic trait group = 0) the clinical cut-off (≥ 70) on the autism measure. All coefficients are unstandardised. ****p* < .001, ***p* < .01, **p* < .05, NS = non-significant.

**References**

Hoekstra, R.A., et al. (2011). The construction and validation of an abridged version of the autism-spectrum quotient (AQ-Short). *Journal of Autism and Developmental Disorders*, *41*(5), 589-596.
